# Feasibility of Point-of-Care Urine Self-Testing to Measure Tenofovir Adherence and Predict Viral Suppression

**DOI:** 10.1093/ofid/ofaf300

**Published:** 2025-05-17

**Authors:** Renata Buccheri, Matthew A Spinelli, Megan J Heise, David V Glidden, Kevin Sassaman, Tyler Martinson, Hannah R Schmidt, Alexa B D’Angelo, Dustin T Duncan, Keith J Horvath, Sabina Hirshfield, Renessa S Williams, Mallory O Johnson, Christian Grov, Adam Carrico, Monica Gandhi

**Affiliations:** Department of Epidemiology and Biostatistics, University of California San Francisco, San Francisco, California, USA; Vitalant Research Institute, San Francisco, California, USA; Division of HIV, Infectious Diseases and Global Medicine, University of California San Francisco, San Francisco, California, USA; Division of HIV, Infectious Diseases and Global Medicine, University of California San Francisco, San Francisco, California, USA; Department of Epidemiology and Biostatistics, University of California San Francisco, San Francisco, California, USA; Division of HIV, Infectious Diseases and Global Medicine, University of California San Francisco, San Francisco, California, USA; Department of Medicine, University of California San Francisco, San Francisco, California, USA; Department of Medicine, University of California San Francisco, San Francisco, California, USA; Department of Community Health and Social Sciences, CUNY Graduate School of Public Health and Health Policy, New York, New York, USA; Department of Epidemiology, Columbia University Mailman School of Public Health, New York City, New York, USA; San Diego State University Department of Psychology, San Diego, California, USA; Department of Medicine, STAR Program, State University of New York—Downstate Health Sciences University, Brooklyn, New York, USA; University of Miami School of Nursing and Health Studies, Coral Gables, Florida, USA; Department of Medicine, Division of Prevention Science, University of California San Francisco, San Francisco, California, USA; City University of New York Graduate School of Public Health, New York, New York, USA; Department of Health Promotion and Disease Prevention, Florida International University, Miami, Florida, USA; Division of HIV, Infectious Diseases and Global Medicine, University of California San Francisco, San Francisco, California, USA

**Keywords:** adherence, antiretroviral therapy, point-of-care monitoring, tenofovir, viral suppression

## Abstract

This study is the first to demonstrate the feasibility of at-home adherence monitoring using a point-of-care urine assay for tenofovir (TFV). Undetectable TFV is strongly associated with lower viral suppression (odds ratio, 0.19, 95% CI, 0.10–0.34; *P* < .001), supporting a streamlined, effective approach for monitoring adherence between viral load assessments.

Only 68% of men who have sex with men (MSM) with HIV have achieved virologic suppression in the United States, which is sufficiently low to align with the goals of the Ending the HIV Epidemic initiative [1]. Viral suppression (VS) is strongly correlated with optimal adherence to antiretroviral therapy (ART) [2]. Approximately 90% of ART regimens worldwide include tenofovir (TFV), co-formulated with lamivudine or emtricitabine. The University of California, San Francisco, Hair Analytical Laboratory developed and validated a low-cost point-of-care (POC) assay to measure urine TFV, providing a reliable adherence metric [3]. This TFV POC assay improved patient management in HIV prevention by enhancing adherence [4] and the accuracy of self-reported PrEP adherence [5]. In clinical and research settings, the POC assay strongly predicts VS [6], and, when combined with adherence counseling, it is associated with improved adherence and VS [4, 7].

This strategy could expand the reach and effectiveness of HIV care adherence monitoring, paving the way for more personalized health care support via telehealth, early recognition of medication adherence challenges, and more effective monitoring and treatment strategies. This study evaluated the correlation between the TFV POC urine assay when self-administered at home and virologic suppression.

## METHODS

This cross-sectional study, conducted over a 1-year period, included a nationally recruited sample of cisgender men with HIV who were aged 18 or older, taking ART, and endorsed challenges with daily ART use. Participants were recruited through a geosocial networking application. Data were stored in REDCap, a HIPAA-compliant database. After completing HIV viral load (VL) measurement at a local commercial phlebotomy site, participants were mailed POC urine TFV kits for self-administration. Urine assay results were submitted to REDCap via secure upload links connected to de-identified participant profiles. Participants were compensated for completing each study procedure (additional details in Supplementary Material 1).

The primary outcome was HIV VL status, dichotomized as suppressed (<200 copies/mL) or unsuppressed (≥200 copies/mL). The primary explanatory variable was the qualitative yes/no result of the POC self-administered urine assay for TFV at a cutoff of 1500 ng/mL, a biomarker indicative of recent ART adherence within the prior 4–7 days [8]. Logistic regression was used to evaluate factors associated with VS, adjusting for demographic and behavioral factors. Although VL was measured first, we assessed the cross-sectional association between self-administered TFV POC assay and VS, the clinical end point of interest in HIV care. Both measures were collected within a narrow time window. The relation between self-reported adherence over the past 30 days, TFV POC results, and VS was assessed by a 2-sample proportion test. Statistical significance was evaluated as *P* < .05. Data analyses were conducted using R (version 4.4.3).

## Patient Consent

The UCSF Institutional Review Board approved all study procedures, and all participants provided informed consent.

## RESULTS

The sample comprised 746 MSM with HIV on TFV-based ART (Table 1). The majority (85%) achieved VS (<200 copies/mL), whereas 15% had unsuppressed VL (≥200 copies/mL). Ninety percent of participants completed and uploaded their urine POC TFV test results. The median time between VL testing and POC testing (interquartile range) was 15 (11–24) days.

**Table 1.** Demographics and Behavioral Characteristics of Participants and Adjusted Logistic Regression Evaluating Factors Associated With Unsuppressed Viral Load

Characteristics	Overall (n = 746), No. (%)	Predictors of VS	
		aOR^a^	95% CI
Age			
Median [min, max], y	40.0 [21.0, 71.7]		
Scaled by 10 y		1.01	0.79–1.30
Race			
White	360 (48)	Ref.	
Black	299 (40)	0.45**	0.25–0.80
Asian	22 (3)	5.32	0.57–135
Other	54 (7)	2.62	0.65–18.00
Not reported	11 (2)		
Hispanic/Latine			
Non-Hispanic	581 (78)	Ref.	
Hispanic	156 (21)	0.81	0.40–1.66
Not reported	9 (1)		
US born			
US born	649 (87)	Ref.	
Not US born	95 (13)	1.02	0.37–2.55
Not reported	2 (0)		
Stimulant use in the past 3 mo			
No use	286 (38)	Ref.	
Occasional use: 1–2 times or monthly	133 (18)	0.61	0.28–1.32
Frequent use: weekly or daily	318 (43)	0.84	0.43–1.62
Not reported	9 (1)		
Viral load testing in past 6 mo			
No	92 (12)	Ref.	
Yes	651 (87)	2.11	0.96–4.54
Missing	2 (0)		
HIV appointment in past 6 mo			
No	72 (10)	Ref.	
Yes	672 (90)	1.04	0.41–2.56
Missing	2 (0)		
Self-report adherence in past month			
Very poor	51 (7)		
Poor	54 (7)		
Fair	107 (14)		
Good	145 (19)		
Very good	221 (30)		
Excellent	165 (22)		
Missing	3 (0)		
Increase by 1 point in adherence		2.33***	1.92–2.85
Urine POC result			
TFV detected	563 (76)	Ref.	
TFV not detected	110 (15)	0.19***	0.10–0.34
TFV assay not returned	73 (10)	0.35**	0.17–0.75
ART regimen			
TAF-based	680 (91)		
TDF-based	66 (9)		
Lab-based viral load			
Suppressed (<200 copies/mL)	631 (85)		
Unsuppressed (≥200 copies/mL)	115 (15)		

Abbreviations: aOR, adjusted odds ratio; ART, antiretroviral therapy; POC, point-of-care; TAF, tenofovir alafenamide; TDF, tenofovir disoproxil fumarate; TFV, tenofovir; VS, viral suppression.

^a^aORs were adjusted for age, race, ethnicity, US-born status, self-report adherence, POC adherence, stimulant use, prior blood draw for viral load in the past 6 months, and prior appointment with an HIV provider in the past 6 months.

*P < .05; **P < .01; ***P < .001.

Both undetectable TFV (adjusted OR [AOR], 0.19; 95% CI, 0.10–0.34; *P* < .001) and not returning the POC assay results (AOR, 0.35; 95% CI, 0.17–0.75; *P* = .006) were associated with decreased odds of VS compared with detectable TFV (Table 1). Among participants with detectable TFV, 92% had VS, compared with 56% in those without detectable TFV (95% CI difference between groups, 26%–46%; *P* < .001). The assay exhibited high sensitivity of 89% and positive predictive value (PPV) of 92%, moderate specificity of 52%, and low negative predictive value (NPV) of 44% (Supplementary Table 1). Self-report adherence, in which participants rated their adherence in the past 30 days on a 6-point scale, was also associated with VS (AOR, 2.36; 95% CI, 1.92–2.95; *P* < .001). Among participants who rated their adherence as “very good” or “excellent,” those with detectable TFV had 98% VS vs 78% without detectable TFV (*P* < .001). Participants rating their adherence as “fair” or “good” with detectable TFV showed 90% suppression, compared with 64% when TFV was undetectable (*P* < .001). For those self-rating “very poor” or “poor,” VS was 62% with detectable TFV and 26% without (*P* = .001) (Supplementary Figure 1). In addition, Black participants had significantly lower rates of VS compared with White participants (AOR, 0.41; 95% CI, 0.22–0.76; *P* = .008).

## DISCUSSION

Our study is the first to establish the feasibility of at-home ART adherence monitoring with the first POC urine-based assay for tenofovir. Undetectable TFV in urine was strongly associated with low VS in this study, exhibiting high sensitivity and PPV. These results indicate that the test could provide a convenient method for self-monitoring adherence between routine VL assessments. This is particularly valuable in minoritized populations, resource-limited settings, or when more frequent monitoring is necessary, such as among those reporting adherence challenges. The test could also be efficient for remote monitoring in the context of telehealth. Relatedly, observed disparities in VS—particularly among Black individuals—emphasize the need for adherence interventions that can help address structural inequities and improve outcomes.

Home-based TFV POC self-administration strongly correlated with VS, mirroring its performance when administered by research staff in a clinical setting [6]. With high acceptability when counseling is performed in a nonjudgmental manner [5], this at-home testing approach could improve HIV outcomes by providing a convenient, effective option for self-monitoring and remotely delivered interventions. Beyond its potential scalability, at-home self-testing offers advantages over POC testing in clinical settings. It provides an accessible option for those unable or unwilling to visit clinical facilities and may help reduce stigma, potentially leading to more consistent monitoring. Home-based testing removes the need for private spaces in clinical environments to collect a urine specimen, addressing challenges in resource-limited health care settings.

By enabling patients to self-monitor, the TFV POC assay could facilitate more timely communication with health care providers, potentially leading to earlier detection of adherence challenges and the need for VL testing. This proactive approach is particularly valuable given the assay's characteristics. The lower specificity and NPV of the TFV POC assay in our study likely reflect the higher potency of modern ART, which permits intermittent adherence without loss of VS [9]. While a negative result may not definitively indicate that a patient is not currently virologically suppressed, the individual is at higher risk of losing VS in the future [6, 10]. Specificity may improve with repeated TFV and VL measurements over time, offering a clearer picture of sustained nonadherence and virologic failure. Furthermore, the combined interpretation of POC TFV results and VL results offers valuable insights into adherence patterns and potential treatment challenges. For instance, low TFV levels in the presence of VS may indicate declining adherence and/or risk of future virologic suppression loss [10], calling for closer monitoring and prophylactic adherence support.

The TFV POC assay can provide valuable insights into potential HIV drug resistance (HIVDR) scenarios. In settings with a high prevalence of HIVDR, particularly among populations failing non-nucleoside reverse transcriptase inhibitor (NNRTI)–based regimens, the TFV POC urine assay has proven to be a reliable predictor of HIVDR in patients with VL >1000 copies/mL [11]. Additionally, among patients using dolutegravir-based regimens, the consistent presence of TFV in urine during viral rebound (VL ≥200 copies/mL) suggests treatment-emergent resistance [7, 10]. By identifying cases where TFV is consistently detectable but VS is not achieved, health care providers can prioritize patients for genotypic resistance testing. This targeted approach could lead to more cost-efficient use of resistance testing and enable timely treatment adjustments.

Previous studies indicate that objective adherence monitoring outperforms self-assessment [11, 12], although self-report is more accurate when participants know their adherence will be objectively assessed [5]. However, even with this awareness, our study still noted discrepancies. For example, 22% who self-rated their past-month adherence as “very good” or “excellent” had undetectable TFV and unsuppressed VL. Conversely, 62% reporting “very poor” or “poor” adherence achieved VS and had a detectable TFV level. Both subjective over- and underestimation of adherence stress the importance of incorporating objective monitoring tools to refine our understanding of perceived, reported, and actual medication adherence.

This study has several limitations. First, it exclusively sampled cisgender sexual minority men via a geosocial networking app, limiting the generalizability to other populations. Second, self-administered tests with participant-uploaded results may introduce measurement bias and pose scalability challenges given the requirement of manual reviews of test results by staff. The potential for automated review of results, potentially using artificial intelligence or image processing, should be studied. Third, although adjustments were made for demographic and behavioral factors, unmeasured confounders may still influence the results. Finally, the reliance on a 1-time nonsimultaneous assessment of TFV and VL within a short time window limits the ability to establish longitudinal patterns or precise temporal alignment.

In summary, we demonstrate the potential of self-administered POC urine TFV testing to enhance HIV treatment adherence monitoring. By providing a reliable, at-home method for ART adherence, this approach could contribute to more effective HIV management strategies.

## Supplementary Material

ofaf300_Supplementary_Data

## Supplementary Data

Supplementary materials are available at *Open Forum Infectious Diseases* online. Consisting of data provided by the authors to benefit the reader, the posted materials are not copyedited and are the sole responsibility of the authors, so questions or comments should be addressed to the corresponding author.
