# Supplementary material for: Feasibility of Point-of-Care Urine Self-Testing to Measure Tenofovir Adherence and Predict Viral Suppression
Source: Open Forum Infect Dis. 2025 May 17;12(6):ofaf300. doi: 10.1093/ofid/ofaf300 (PMC12131155; doi:10.1093/ofid/ofaf300)
Supplement: ofaf300_Supplementary_Data [file ofaf300_supplementary_data.zip › SupplementaryData_April2025.docx]

**Supplementary Material S1.** Additional Methodological Details

Participants were recruited through a geosocial networking application via advertisements that directed users to a secure pre-screener survey. Data were stored in a HIPAA-compliant database (e.g., REDCAP), and identifying information was retained in order to mail participants study materials. After completing HIV viral load (VL) measurement at a local commercial phlebotomy site, participants were mailed POC urine TFV kits for self-administration. This commercial phlebotomy site has a research division to coordinate transferring participants’ VL results while maintaining confidentiality. Urine assay results were submitted to the REDCAP database via secure upload links using unique study identifiers. Participants were compensated for completing each study procedure (i.e., viral load, completion of surveys, submitting a photo of POC test).

**Supplementary Table 1:** Urine Tenofovir Point Of Care Results and HIV Viral Load Status

| **Method** | **Category** | **Viral Load** | | **Metrics** | | | | |
| --- | --- | --- | --- | --- | --- | --- | --- | --- |
|  |  | **Suppressed** | **Unsuppressed** | **Sensitivity** | **Specificity** | **PPV** | **NPV** | **Accuracy** |
|  |  | **<200cp/mL** | **≥200 cp/mL** | **(95%CI)** | **(95%CI)** | **(95%CI)** | **(95%CI)** | **(95%CI)** |
| **Urine TFV POC Result (n=663)** | TFV Detected | 519 (92%) | 44 (8%) | 0.89 | 0.52 | 0.92 | 0.44 | 0.84 |
|  | TFV Not Detected | 62 (56%) | 48 (44%) | (0.87-0.92) | (0.42-0.63) | (0.90-0.94) | (0.34-0.53) | (0.81-0.87) |

Abbreviation: TFV, tenofovir; POC, point-of-care; HIV, human immunodeficiency virus; CI, confidence interval; PPV, positive predictive value; NPV, negative predictive value.

**
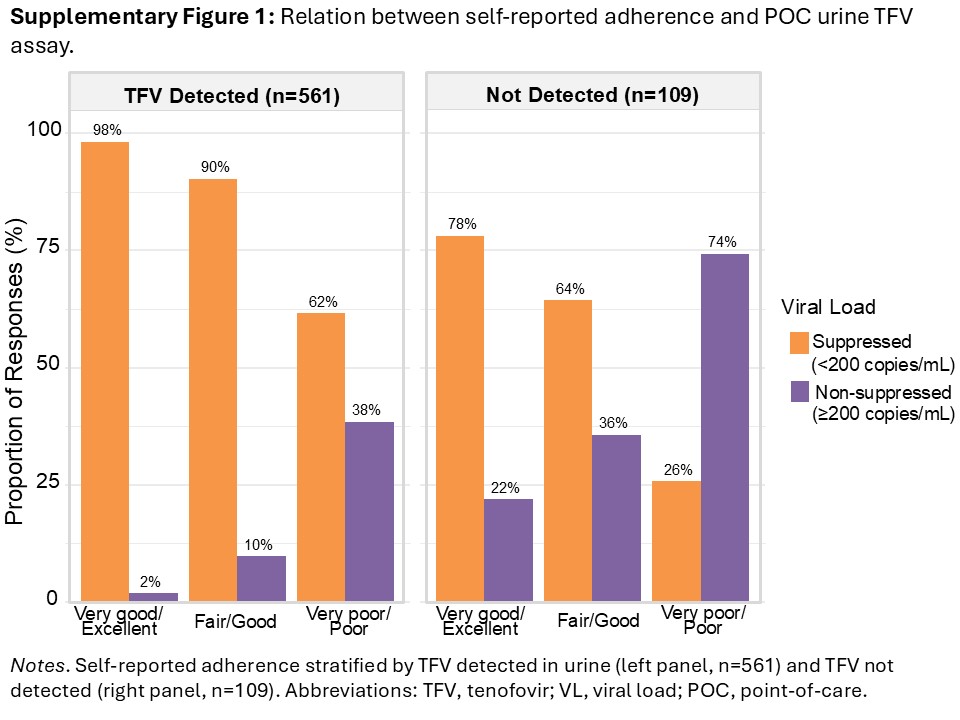
**

We depict bar charts comparing self-reported adherence rates in the past 30 days, stratified by viral load (VL) in two groups according to the point-of-care (POC) urine tenofovir (TFV) result. The left panel represents participants with TFV detected in urine (n=561), and the right panel represents those without TFV detected (n=109). Analysis excluded n=76 participants missing the POC urine assay and/or self-report adherence in the past 30 days. Adherence categories include "Very Good" or "Excellent," "Fair" or "Good," and "Very Poor" or "Poor." Viral load status is color-coded: orange for VL <200 copies/mL and purple for VL ≥200 copies/mL. In the TFV-detected group, most participants who rated their adherence as "Very Good" or "Excellent" had a suppressed VL (<200 copies/mL), while those with "Very Poor" or "Poor" adherence had a higher proportion of participants with VL ≥200 copies/mL. In the TFV-not-detected group, the "Very Poor" or "Poor" adherence category had a greater proportion of participants with VL ≥200 copies/mL. The TFV-detected group excludes two unknown values, and the TFV-not-detected group excludes one.
