# Supplementary figures and images for: Feasibility of Point-of-Care Urine Self-Testing to Measure Tenofovir Adherence and Predict Viral Suppression
Source: Open Forum Infect Dis. 2025 May 17;12(6):ofaf300. doi: 10.1093/ofid/ofaf300 (PMC12131155; doi:10.1093/ofid/ofaf300)

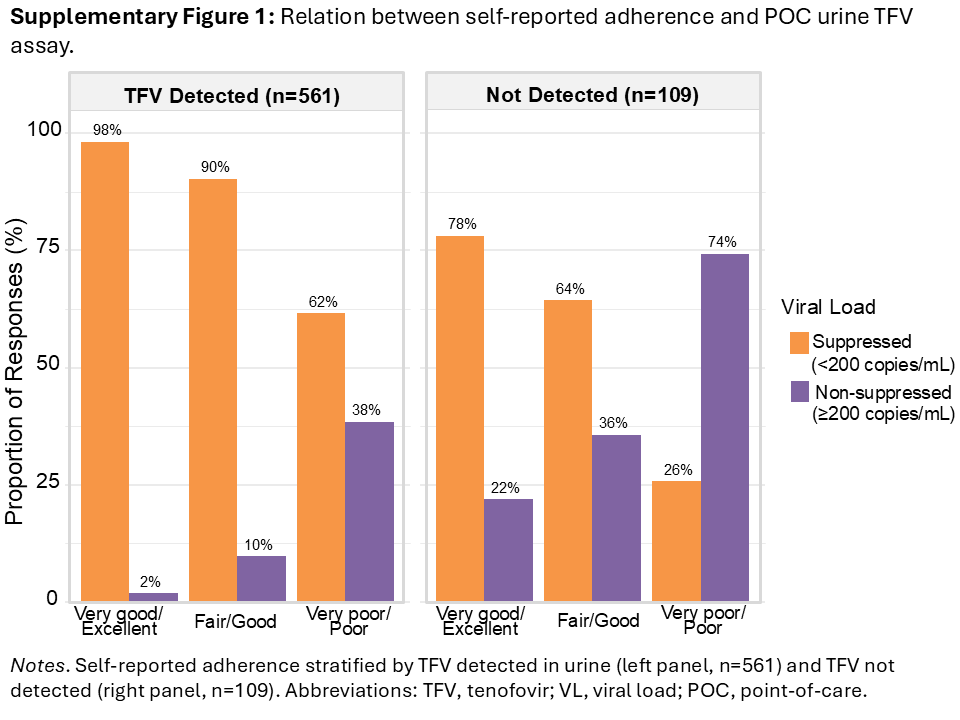

Supplement: ofaf300_Supplementary_Data [file ofaf300_supplementary_data.zip › Supplementary_figure1_600.tif]
